# Antioxidant, Anti-Inflammatory, and Bone-Remodeling Modulatory Activities of Thai Pigmented and Non-Pigmented Rice Bran Oils Containing Tocols and γ-Oryzanol

**DOI:** 10.3390/antiox15080981

**Published:** 2026-08-07

**Authors:** Thanawat Pattananandecha, Sutasinee Apichai, Jakaphun Julsrigival, Kannika Thiankhanithikun, Fumihiko Ogata, Naohito Kawasaki, Jetsada Ruangsuriya, Chalermpong Saenjum

**Affiliations:** 1 Office of Research Administration, Chiang Mai University, Chiang Mai 50200, Thailand; sutasinee.apichai@cmu.ac.th; 2 Research Center for Innovation in Analytical Science and Technology for Biodiversity-Based Economic and Society (I-ANALY-S-T_B.BES-CMU), Chiang Mai University, Chiang Mai 50200, Thailand; jakaphun.jul@cmu.ac.th (J.J.); kannika.th@cmu.ac.th (K.T.); jetsada.ruang@cmu.ac.th (J.R.); 3 Department of Pharmaceutical Sciences, Faculty of Pharmacy, Chiang Mai University, Chiang Mai 50200, Thailand; 4 Department of Pharmaceutical Care, Faculty of Pharmacy, Chiang Mai University, Chiang Mai 50200, Thailand; 5 Faculty of Pharmacy, Kindai University, 3-4-1 Kowakae, Higashi-Osaka 577-8502, Osaka, Japan; ogata@phar.kindai.ac.jp (F.O.); kawasaki@phar.kindai.ac.jp (N.K.); 6 Antiaging Center, Kindai University, 3-4-1 Kowakae, Higashi-Osaka 577-8502, Osaka, Japan; 7 Department of Biochemistry, Faculty of Medicine, Chiang Mai University, Chiang Mai 50200, Thailand

**Keywords:** Thai rice bran oil, tocopherols, tocotrienols, oxidative stress, inflammation, osteoblasts, bone remodeling

## Abstract

Rice bran oil (RBO) is a rich source of lipophilic bioactive compounds that may contribute to antioxidant, anti-inflammatory, and bone-health-promoting activities. This study investigated the effects of RBOs containing tocopherols, tocotrienols, and γ-oryzanol derived from Thai pigmented and non-pigmented rice cultivars on oxidative stress, inflammatory responses, and bone-remodeling-related activities. The content of tocopherols, tocotrienols, and γ-oryzanol was determined by high-performance liquid chromatography (HPLC). Reactive oxygen species (ROS) production was determined in human fetal osteoblast (hFOB 1.19) cells where nitric oxide (NO) and inducible nitric oxide synthase (iNOS) production were evaluated in RAW264.7 cells. Following compositional profiling and correlation analyses of antioxidant and anti-inflammatory activities, three representative RBOs were subsequently evaluated for their effects on bone-remodeling-related activities in hFOB 1.19 cells through alkaline phosphatase (ALP) activity and the production of osteocalcin (OC), osteoprotegerin (OPG), and receptor activator of nuclear factor kappa-B ligand (RANKL). Additionally, calcification was investigated by Alizarin Red S staining. Pigmented RBOs, particularly Khao’ Hom Nil and RD69, contained higher levels of tocopherols, tocotrienols, and γ-oryzanol than non-pigmented RBOs. These oils significantly suppressed ROS, NO, and iNOS production, which correlated with tocopherol, tocotrienol, and γ-oryzanol content, while enhancing ALP activity, OC and OPG production, reducing RANKL levels and the RANKL/OPG ratio, and promoting matrix mineralization. Overall, pigmented RBOs represent promising natural sources of lipophilic bioactive compounds with potential antioxidant, anti-inflammatory, and bone-remodeling-related activities under in vitro conditions.

## 1. Introduction

Bone remodeling is a dynamic and tightly regulated process that maintains skeletal integrity through the balance between osteoblast-mediated bone formation and osteoclast-driven bone resorption [1,2]. Disruption of this balance, particularly under conditions of oxidative stress and chronic inflammation, contributes to the development of skeletal disorders such as osteoporosis and inflammatory bone loss [3,4,5]. Pro-inflammatory mediators, including tumor necrosis factor-α (TNF-α) and receptor activator of nuclear factor kappa-B ligand (RANKL), are known to promote osteoclastogenesis through signaling pathways such as NF-κB [3,5,6]. In parallel, excessive production of reactive oxygen species (ROS) and reactive nitrogen species (RNS) has been shown to impair osteoblast function and enhance osteoclast activity, further accelerating bone deterioration [7,8].

Natural bioactive compounds with antioxidant and anti-inflammatory properties have attracted increasing attention as potential modulators of bone remodeling [9,10,11]. Rice (*Oryza sativa* L.) is one of the most important staple crops worldwide [12]. In Thailand, rice comprises diverse varieties, including pigmented rice such as red and purple rice [13,14]. These pigmented varieties possess distinct phytochemical profiles compared with non-pigmented rice, which may result in differences in biological activities, particularly antioxidant and anti-inflammatory effects [13,15,16,17]. Pigmented rice has also been reported to contain higher levels of lipid-soluble bioactive compounds, particularly tocopherols, tocotrienols, and γ-oryzanol, than non-pigmented rice [18,19].

Rice bran oil (RBO) is enriched primarily in lipid-soluble bioactive compounds, particularly tocopherols, tocotrienols, and γ-oryzanol [20,21,22], which have been widely reported to possess antioxidant and anti-inflammatory activities [20,23,24]. These compounds have also been associated with osteoblast activity and bone remodeling: tocotrienols enhance osteoblast activity and suppress osteoclast differentiation [25,26,27,28], while γ-oryzanol has also been reported to support osteoblast activity and bone formation-related processes [29,30]. However, to the best of our knowledge, no study has systematically compared white, red, and purple Thai RBOs by integrating phytochemical characterization with in vitro antioxidant, anti-inflammatory, and bone-remodeling-related evaluations. Furthermore, the relationships between the major lipophilic bioactive compounds and the observed cell-based biological activities remain insufficiently characterized.

Therefore, the present study aimed to systematically compare RBOs derived from representative white, red, and purple Thai rice cultivars by integrating phytochemical analysis with cell-based biological evaluations. The major lipophilic bioactive compounds, including tocopherols, tocotrienols, and γ-oryzanol, were first determined, and their relationships with antioxidant and anti-inflammatory activities were explored through correlation analysis. Antioxidant activity was evaluated by measuring intracellular ROS production in human fetal osteoblast (hFOB 1.19) cells, while anti-inflammatory activity was evaluated by determining nitric oxide (NO) and inducible nitric oxide synthase (iNOS) production in RAW264.7 macrophages. Selected representative RBOs were subsequently evaluated for their effects on bone-remodeling-related markers, including alkaline phosphatase (ALP) activity and the production of osteocalcin (OC), osteoprotegerin (OPG), and RANKL in hFOB 1.19 cells. Furthermore, matrix mineralization was evaluated by Alizarin Red S staining to provide complementary evidence of osteogenic potential in vitro.

## 2. Materials and Methods

### 2.1. Reagents and Chemicals

HPLC-grade methanol (LC1115), acetonitrile (LC1005), ethanol (AR1380), dichloromethane (LC1040A), and sodium hydroxide (AR1171) were obtained from RCI Labscan Ltd. (Bangkok, Thailand). Tocopherol standards (α-, β-, γ-, and δ-tocopherol; Tocopherol Set, 613424) and tocotrienol standards (α-tocotrienol, 07205; β-tocotrienol, 05644; γ-tocotrienol, 49634; and δ-tocotrienol, 69745), L-ascorbic acid (A4403), N-acetylcysteine (NAC; A9165), 2′,7′-dichlorodihydrofluorescein diacetate (H_2_DCF-DA; D6883), curcumin (C1386), Alizarin Red S (K48040178), β-glycerophosphate (G5422), acetic acid (K53439863-121), dimethyl sulfoxide (DMSO; D8418), Bradford reagent (B6916), bovine serum albumin (BSA) standard (A9418), and CellLytic™ M Cell Lysis Buffer (C2978), were purchased from Sigma-Aldrich (St. Louis, MO, USA). The γ-oryzanol standard (154-01271) was purchased from FUJIFILM Wako Pure Chemical Corporation (Osaka, Japan). Dulbecco’s Modified Eagle Medium (DMEM)/F-12 without phenol red (11039021), Geneticin selective antibiotic (G418 sulfate; 10131035), fetal bovine serum (FBS; A5256701), trypsin–EDTA solution (15400054), phosphate-buffered saline (PBS, pH 7.4; 10010023), and PrestoBlue™ Cell Viability Reagent (A13261) were supplied by Thermo Fisher Scientific (Waltham, MA, USA). Human Osteocalcin ELISA Kit (AB270202), Human Osteoprotegerin ELISA Kit (AB189580), and Human TNFSF11 (RANKL) ELISA Kit (AB213841) were obtained from Abcam (Cambridge, UK), whereas the Mouse iNOS ELISA Kit (CSB-E08326M) was purchased from Cusabio Biotech (Houston, TX, USA). *p*-Nitrophenyl phosphate (pNPP; A12310), *p*-nitrophenol (pNP; 73560-100G), dexamethasone (1A 1347/30), and diethanolamine (00098) were purchased from Alfa Aesar (Haverhill, MA, USA), Fluka (Buchs, Switzerland), L.B.S. Laboratory Co. Ltd. (Bangkok, Thailand), and Loba Chemie Pvt. Ltd. (Mumbai, India), respectively.

### 2.2. Rice Bran Samples

Rice bran samples, Khao’ Dawk Mali 105 (KMDL105), RD61, and RD41 were classified as white rice bran; RD69 (Tubtim Chumphae), Red Jasmin Rice, and Sang Yod were classified as red rice bran; and Khao’ Hom Nil, Riceberry, and CMU107 were classified as purple rice bran. All rice bran samples were obtained from rice cultivated in Thailand through the Rice Research Center in Thailand. The rice bran samples were dried at 60 °C for 12 h prior to milling, and the resulting rice bran was passed through a 60-mesh sieve and subjected to enzyme deactivation at 60 °C for 1 h before extraction.

### 2.3. Preparation of RBOs

RBOs were prepared using a laboratory-scale supercritical CO_2_ extraction system (SFE Lab, SFE Process, Villers-lès-Nancy, France) according to a previously reported method with modifications [31]. Briefly, 50 g of enzyme-deactivated rice bran from each cultivar was loaded into the extraction vessel. Supercritical CO_2_ extraction was performed at 60 °C, 450 bars for 120 min of extraction at a constant CO_2_ flow rate of 5.0 g/min. Each cultivar was extracted in triplicate. The obtained RBOs were collected, weighed to determine the extraction yield, and stored at −20 °C until further analysis.

### 2.4. Determination of Major Bioactive Compounds

#### 2.4.1. Tocopherols and Tocotrienols

Tocopherol and tocotrienol content in RBOs was determined using reversed-phase high-performance liquid chromatography (RP-HPLC) according to our previously reported method with slight modifications [32]. The analysis was performed using an Agilent 1200 HPLC system (Agilent Technologies, Santa Clara, CA, USA) equipped with a fluorescence detector. Separation was achieved using a Kinetex^TM^ PFP column (150 × 4.6 mm, 2.6 µm; Phenomenex, Torrance, CA, USA) maintained at 30 °C. Methanol and water (9:1, *v*/*v*) were used as the mobile phase at a flow rate of 0.6 mL/min. The excitation and emission wavelengths were set at 292 and 330 nm, respectively. The injection volume was 10 µL. For sample preparation, 10 mg of each RBO sample was dissolved in 1 mL methanol and filtered through a 0.22 µm syringe filter prior to analysis. Quantification was performed using external standards of α-, β-, γ-, and δ-tocopherols and α-, β-, γ-, and δ-tocotrienols. All samples were analyzed in triplicate.

#### 2.4.2. γ-Oryzanol

The γ-oryzanol content was determined by HPLC with slight modifications of a previously reported method [31]. The analysis was performed using the same Agilent 1200 HPLC system equipped with a UV detector set at 330 nm. Separation was achieved on a Symmetry C18 column (250 × 4.6 mm, 5 µm; Waters, Milford, MA, USA) maintained at 30 °C. The mobile phase consisted of methanol, acetonitrile, dichloromethane, and acetic acid in the ratio of 50:44:3:3 *v*/*v* at a flow rate of 1.0 mL/min. The injection volume was 10 µL. Quantification was performed using an external γ-oryzanol standard. All samples were analyzed in triplicate. Total γ-oryzanol was calculated as the sum of cycloartenyl ferulate, 24-methylenecycloartanyl ferulate, campesteryl ferulate, and β-sitosteryl ferulate.

The analytical performance of the two HPLC methods, including linear ranges, linearity (R^2^), limits of detection (LOD), and limits of quantification (LOQ) for the representative analytes, is summarized in Supplementary Table S2.

### 2.5. Cell Culture and Cytotoxicity Assay

Murine macrophage cells (RAW264.7; ATCC, Manassas, VA, USA; TIB-71™; CVCL_0493) were used as an in vitro model for evaluating anti-inflammatory activity. Cells were cultured in DMEM supplemented with 10% FBS under a humidified atmosphere of 5% CO_2_ at 37 °C. Human fetal osteoblast cells (hFOB 1.19, ATCC, Manassas, VA, USA; CRL-11372™; CVCL_3708) were used as an in vitro model to evaluate ROS production and bone-remodeling-related parameters. Cells were cultured in DMEM/Ham’s F-12 medium supplemented with 10% FBS and 0.3 mg/mL Geneticin under a humidified atmosphere of 5% CO_2_ at 37 °C, following the culture conditions previously described for evaluating osteoblast function-related markers in hFOB 1.19 cells [33].

For cytotoxicity evaluation, RAW264.7 and hFOB 1.19 cells were seeded into 96-well plates at a density of 1 × 10^4^ cells per well and allowed to adhere overnight. RBOs were dissolved in 100% DMSO and subsequently diluted with the respective complete culture media. Cells were treated with RBOs at final concentrations of 25, 50, 100, and 200 µg/mL. The final concentration of DMSO did not exceed 0.5% (*v*/*v*) in any treatment group, and cells treated with the corresponding concentration of DMSO served as the vehicle control. After 72 h of incubation, the treatment medium was removed, and fresh medium containing 10% (*v*/*v*) PrestoBlue^TM^ Cell Viability Reagent (Thermo Fisher Scientific, Waltham, MA, USA) was added to each well. Cell viability was then determined according to the manufacturer’s instructions.

### 2.6. The Effect of RBOs on Intracellular ROS and RNS Production

#### 2.6.1. Intracellular ROS Production Assay

Intracellular ROS production was determined using H_2_DCF-DA as a fluorescent probe, following a slightly modified method as previously reported [34]. Briefly, hFOB 1.19 cells were seeded into 96-well plates (1 × 10^4^ cells per well) and pretreated with RBOs (100 and 200 µg/mL), or NAC (50 µM), or L-ascorbic acid (6.25 and 12.5 µg/mL) as positive antioxidant controls for 24 h. Oxidative stress was induced by exposure to 100 µM H_2_O_2_ for 1 h, followed by incubation with 40 µM H_2_DCF-DA at 37 °C for 30 min in the dark. Fluorescence intensity was measured using a microplate reader at excitation and emission wavelengths of 480 and 525 nm, respectively.

#### 2.6.2. Inhibitory Effects on NO and iNOS Production

The inhibitory effects of RBOs on NO and iNOS production in RAW264.7 cells were determined as previously described [35]. Briefly, RAW264.7 cells were seeded into 24-well plates (1 × 10^5^ cells per well) and cultured in DMEM supplemented with 10% FBS, 100 U/mL penicillin, and 100 µg/mL streptomycin under a humidified atmosphere containing 5% CO_2_ at 37 °C. The cells were pretreated with RBOs (100 and 200 µg/mL) for 12 h, followed by stimulation with LPS (2 ng/mL) and IFN-γ (50 pg/mL) for 72 h, to induce inflammatory responses in the RAW264.7 cells [36]. Unstimulated cells served as the negative control, whereas LPS/IFN-γ-stimulated cells served as the inflammatory control. Curcumin (2.5 µg/mL) was used as the positive anti-inflammatory control. NO production was determined by measuring nitrite levels in the culture supernatants using Griess reagent at 540 nm. Nitrite concentrations were calculated using a potassium nitrite standard curve. The iNOS levels in cell lysates were determined using a mouse iNOS ELISA kit. Cell lysates were prepared using CelLytic^TM^ M Cell Lysis Buffer (Sigma-Aldrich, St. Louis, MO, USA) according to the manufacturer’s instructions.

### 2.7. The Effects of Representative RBOs on Bone-Remodeling-Related Parameters

The effects of representative RBOs on bone-remodeling-related parameters were evaluated using hFOB 1.19 cells. Based on the cytotoxicity assay and the antioxidant and anti-inflammatory screening results, RBO concentrations of 25, 50, 100, and 200 µg/mL were selected for subsequent experiments. Cells were seeded in 24-well plates at a density of 1.0 × 10^5^ cells per well and cultured in culture medium containing the indicated concentrations of RBOs for 7 and 14 days under a humidified atmosphere containing 5% CO_2_ at 37 °C. After treatment, cell lysates were collected using CelLytic™ M Cell Lysis Buffer for ALP determination, and culture supernatants were harvested for the determination of bone remodeling markers, including OC, OPG, and RANKL.

#### 2.7.1. ALP Activity

ALP activity was determined according to a previously reported method with slight modifications [34]. Briefly, cell lysates were incubated with pNPP in diethanolamine buffer (pH 9.8) at 37 °C for 120 min. The reaction was terminated by the addition of NaOH, and the absorbance was measured at 405 nm. ALP activity was calculated from the amount of pNP produced and normalized to the total protein content. Total protein content was determined using the Bradford assay. Samples were mixed with Bradford reagent, incubated at room temperature for 5 min, and the absorbance was measured at 595 nm. Protein concentrations were calculated using a BSA standard curve.

#### 2.7.2. Determination of OC, OPG, and RANKL Concentrations

The concentration of OC, OPG, and RANKL in culture supernatants was quantified using commercial SimpleStep ELISA^®^ kits (Abcam, Cambridge, UK) according to the manufacturer’s instructions. Briefly, samples for OC and OPG analysis were incubated for 1 h 30 min, whereas those for RANKL analysis were incubated for 3 h 30 min. Absorbance was measured at 450 nm, and concentrations were calculated from standard curves and expressed in pg/mL or ng/mL, as appropriate. To further evaluate the balance between bone formation and resorption, the OPG/RANKL ratio was calculated.

### 2.8. Alizarin Red S Staining

Matrix mineralization was evaluated using Alizarin Red S staining according to a previously reported method with slight modifications [37]. Briefly, hFOB 1.19 cells were seeded at a density of 1.0 × 10^4^ cells per well in DMEM/F-12 medium and allowed to attach for 24 h. The culture medium was then replaced with osteogenic differentiation medium consisting of DMEM/F-12 supplemented with 10 mM β-glycerophosphate, 0.01 µM dexamethasone, and 0.2 mM L-ascorbic acid. Based on the results of the bone-remodeling-related assays, the selected representative RBOs were further evaluated at 100 and 200 µg/mL. Cells cultured in osteogenic differentiation medium without RBOs treatment served as the control. The medium was replaced every 3 days, and cells were incubated for 14 or 21 days to capture the later-stage progression of matrix mineralization.

Following incubation, the cells were washed with PBS, fixed with 50% ethanol for 15 min at 4 °C, rinsed with deionized water, and stained with Alizarin Red S solution for 30 min at room temperature. Mineralized nodules were observed under an inverted microscope (Motic AE2000, Kowloon, Hong Kong), and representative images were captured. For quantitative analysis, the bound dye was extracted using 10% (*v*/*v*) acetic acid in deionized water/methanol (8:2, *v*/*v*), and the absorbance was measured at 405 nm using a SpectraMax M3 microplate reader (Molecular Devices, San Jose, CA, USA).

### 2.9. Statistical Analysis

Statistical analyses were performed using SPSS version 25.0 (IBM Corporation, Armonk, NY, USA) and GraphPad Prism version 10.4.2 (GraphPad Software, San Diego, CA, USA). For cell-based experiments, data are presented as the mean ± SD of three independent biological experiments (*n* = 3). Differences between multiple groups in the cytotoxicity assay were analyzed using one-way analysis of variance (ANOVA) followed by Dunnett’s multiple comparisons test. Cell-based experiments were analyzed using two-way ANOVA followed by Dunnett’s multiple comparisons test for comparisons with the corresponding control group and Tukey’s multiple comparisons test for comparisons between RBO cultivars. For intracellular ROS, NO, and iNOS assays, Šídák’s multiple comparisons test was additionally performed to compare the 100 and 200 µg/mL treatments within the same RBO cultivar. Pearson correlation analysis was performed using data from the nine RBO samples to evaluate the relationships between total tocopherol, total tocotrienol, and γ-oryzanol contents and the intracellular ROS, NO, and iNOS productions. Statistical significance was set at *p* < 0.05.

## 3. Results

### 3.1. Determination of Major Bioactive Compounds

The extraction yields of RBOs obtained from different rice cultivars are summarized in Table S1. The oil yields ranged from 11.75% to 14.32%, with pigmented rice cultivars generally exhibiting higher extraction yields than non-pigmented cultivars. Tocopherols, tocotrienols, and γ-oryzanol were subsequently quantified by HPLC, and the results are presented below.

#### 3.1.1. Tocopherols and Tocotrienols

The tocopherol and tocotrienol contents of RBOs obtained from different rice varieties is presented in Table 1 and Table 2, respectively. β-Tocotrienol was not detected in any of the RBO samples analyzed. Representative RP-HPLC chromatograms of tocopherol and tocotrienol standards and a representative sample, together with the corresponding peak assignments, are provided in Supplementary Figure S1. Among the tocopherol isomers, γ-tocopherol was the predominant isomer in all RBOs, followed by α-, β-, and δ-tocopherol. Similarly, γ-tocotrienol was the predominant tocotrienol isomer detected in all samples. The total tocopherol and tocotrienol contents varied considerably between the rice varieties, with pigmented RBOs generally containing higher levels than white RBOs. Khao’ Hom Nil RBO exhibited the highest total tocopherol and total tocotrienol content among all samples. Among the white, red, and purple RBOs, KMDL105, RD69, and Khao’ Hom Nil exhibited the highest total tocopherol and tocotrienol contents within their respective groups.

#### 3.1.2. γ-Oryzanol

The γ-oryzanol content of RBOs obtained from different rice varieties is summarized in Table 3. Representative RP-HPLC chromatograms of the γ-oryzanol standard and a representative RBO sample, together with the corresponding peak assignments, are presented in Supplementary Figure S2. The γ-oryzanol content varied between the rice varieties, with pigmented RBOs generally exhibiting higher levels than white RBOs. RD69 contained the highest γ-oryzanol content among all cultivars, followed by Khao’ Hom Nil, Riceberry, and Sang Yod, whereas KMDL105 exhibited the highest γ-oryzanol content among the white RBOs. Overall, the variation in γ-oryzanol content was broadly consistent with the distribution patterns of total tocopherols and tocotrienols across the analyzed rice varieties.

### 3.2. Cytotoxicity of RBOs in RAW264.7 and hFOB 1.19 Cells

The cytotoxicity of RBOs on RAW264.7 and hFOB 1.19 cells was evaluated using the PrestoBlue™ assay. No significant cytotoxicity was observed following treatment with RBOs at concentrations ranging from 25 to 200 µg/mL for 72 h of incubation. Cell viability remained above 80% in both cell lines at all tested concentrations (Supplementary Figure S3), indicating that the tested concentrations were non-cytotoxic under the experimental conditions. Therefore, RBO concentrations of 25 to 200 µg/mL were used in subsequent antioxidant, anti-inflammatory, and bone-remodeling-related assays.

### 3.3. The Effect of RBOs on Intracellular ROS and RNS Production

#### 3.3.1. Intracellular ROS Production Assay

**Figure 1:**
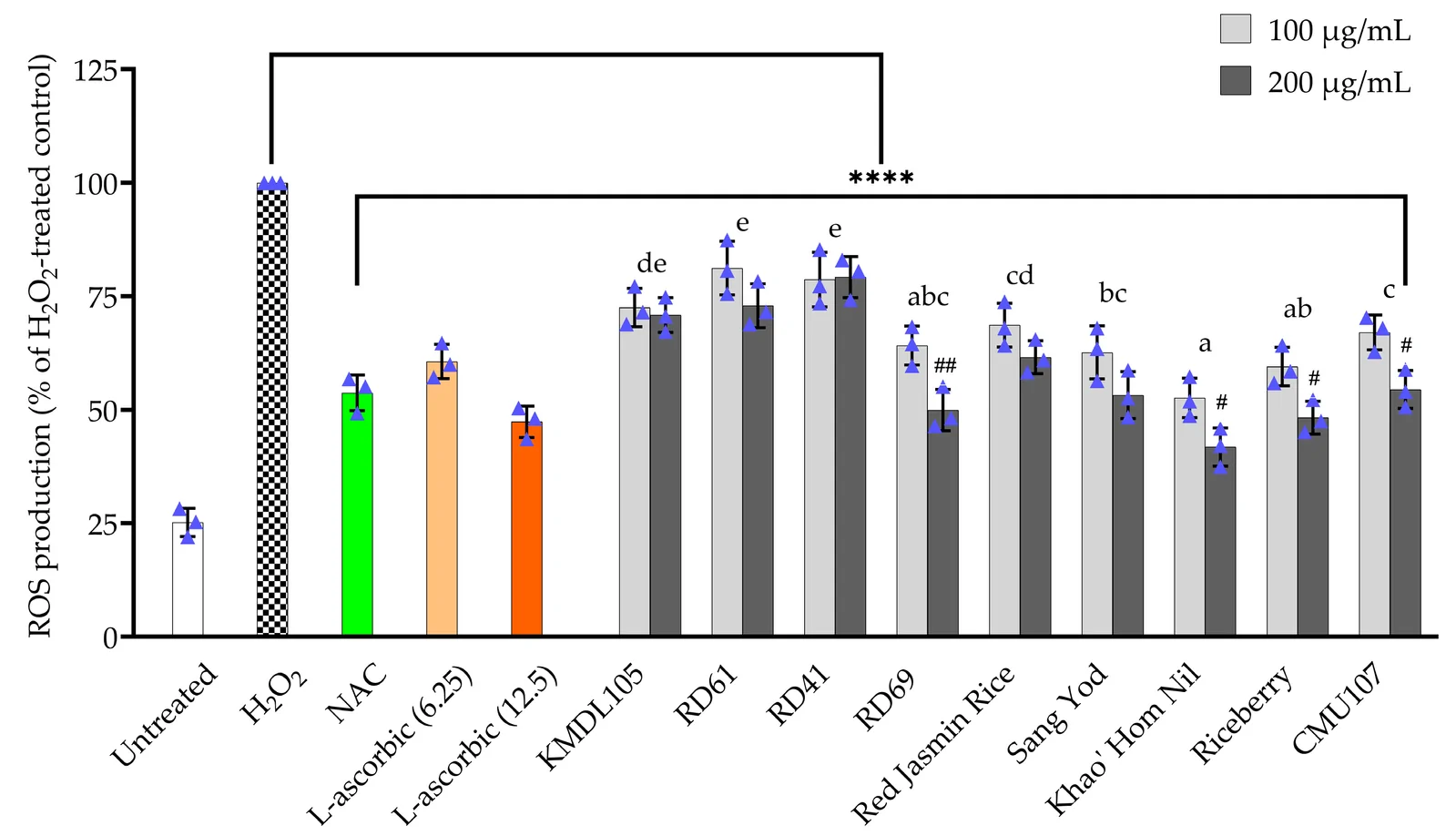
The effects of RBOs (100 and 200 µg/mL) on intracellular ROS production in H_2_O_2_-treated hFOB 1.19 cells. Untreated hFOB 1.19 cells served as the negative control, whereas H_2_O_2_-treated hFOB 1.19 cells served as the oxidative stress control. N-acetylcysteine (NAC, 50 µM) and L-ascorbic acid (6.25 and 12.5 µg/mL) were used as positive antioxidant controls. Data are presented as the mean ± SD (*n* = 3). Blue triangles represent individual data points. Statistical significance was determined by two-way ANOVA followed by Dunnett’s multiple comparisons test for comparisons with the oxidative stress control, Šídák’s multiple comparisons test for comparisons between the 100 and 200 µg/mL treatments of the same RBO, and Tukey’s multiple comparisons test for comparisons between RBO cultivars based on the main effect of cultivar (averaged across both treatment concentrations). Different lowercase letters indicate significant differences between RBO cultivars based on the main effect of cultivar. **** *p* < 0.0001 compared with the oxidative stress control. # *p* < 0.05 and ## *p* < 0.01 compared with the corresponding 100 µg/mL treatment of the same RBO.

The effects of RBOs on intracellular ROS production in H_2_O_2_-treated hFOB 1.19 cells are shown in Figure 1. NAC (50 µM) and L-ascorbic acid (6.25 and 12.5 µg/mL) were used as positive antioxidant controls and significantly reduced ROS production compared to the H_2_O_2_-treated control (*p* < 0.0001). All RBOs significantly suppressed intracellular ROS production at both tested concentrations (*p* < 0.0001); a significantly greater inhibitory effect was observed at 200 µg/mL than at 100 µg/mL for RD69, Khao’ Hom Nil, Riceberry, and CMU107. Among the tested cultivars, Khao’ Hom Nil was among the most effective at suppressing intracellular ROS production.

#### 3.3.2. Inhibitory Effects on NO and iNOS Production

**Figure 2:**
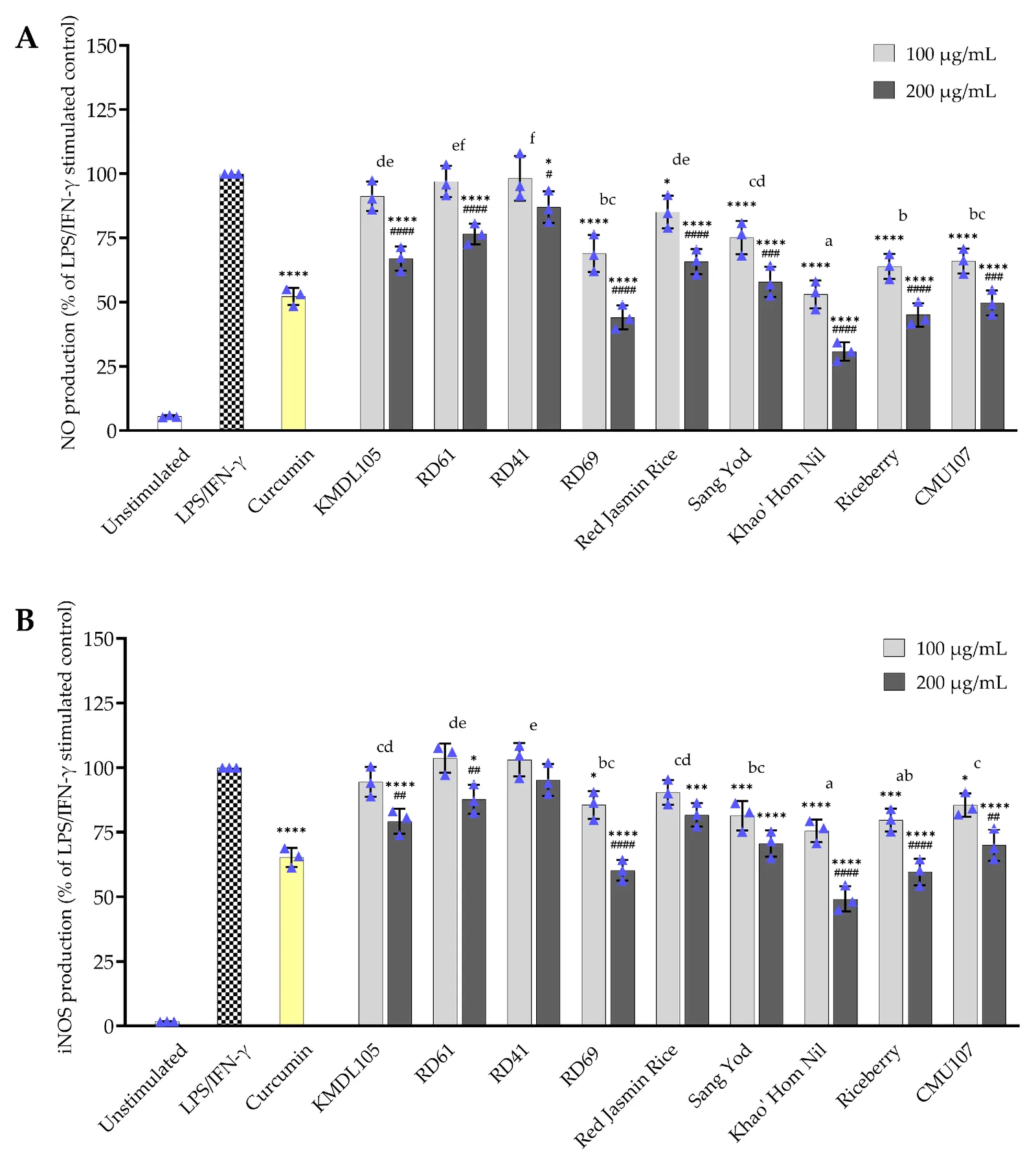
The effects of RBOs (100 and 200 µg/mL) on (**A**) NO and (**B**) iNOS production in LPS/IFN-γ-stimulated RAW264.7 cells. Unstimulated RAW264.7 cells served as the negative control, whereas LPS/IFN-γ-stimulated RAW264.7 cells served as the inflammatory control. Curcumin (2.5 µg/mL) was used as the positive anti-inflammatory control. The data are presented as the mean ± SD (*n* = 3). Blue triangles represent individual data points. Statistical significance was determined by two-way ANOVA followed by Dunnett’s multiple comparisons test for comparisons with the oxidative stress control, Šídák’s multiple comparisons test for comparisons between the 100 and 200 µg/mL treatments of the same RBO, and Tukey’s multiple comparisons test for comparisons between RBO cultivars based on the main effect of cultivar (averaged across both treatment concentrations). Different lowercase letters indicate significant differences between RBO cultivars based on the main effect of cultivar. * *p* < 0.05, *** *p* < 0.001, and **** *p* < 0.0001 compared with the oxidative stress control. # *p* < 0.05, ## *p* < 0.01, ### *p* < 0.001, and #### *p* < 0.0001 compared with the corresponding 100 µg/mL treatment of the same RBO.

The inhibitory effects of RBOs on NO and iNOS production in LPS/IFN-γ-stimulated RAW264.7 cells are shown in Figure 2A and Figure 2B, respectively. Curcumin (2.5 µg/mL), used as the positive anti-inflammatory control, significantly reduced both NO and iNOS production compared with the LPS/IFN-γ-stimulated control (*p* < 0.0001). Treatment with RBOs significantly reduced NO and iNOS production. For NO production, treatment with 200 µg/mL RBO resulted in significantly greater inhibition than treatment with 100 µg/mL across all cultivars. For iNOS production, significantly greater inhibition at 200 µg/mL was observed in five cultivars. Among the tested cultivars, Khao’ Hom Nil exhibited the lowest NO and iNOS production.

#### 3.3.3. Correlation Between Tocols and γ-Oryzanol Content in RBOs on Intracellular ROS and RNS Production

Pearson correlation analysis revealed significant negative correlations between the total tocopherol, total tocotrienol, and γ-oryzanol content of the nine RBO samples and intracellular ROS (*r* = −0.983, −0.987, and −0.946, respectively), NO (*r* = −0.931, −0.958, and −0.886, respectively), and iNOS production (*r* = −0.916, −0.958, and −0.896, respectively) at 200 µg/mL (all *p* < 0.005; Supplementary Table S3). These correlations indicate an association between the lipophilic bioactive constituents and the observed antioxidant and anti-inflammatory activities.

Based on the overall compositional profiles, antioxidant and anti-inflammatory screening results, and supporting statistical analyses, KMDL105, RD69, and Khao’ Hom Nil were selected as representative white, red, and purple RBOs, respectively, for subsequent bone-remodeling-related experiments.

### 3.4. The Effects of Representative RBOs on Bone-Remodeling-Related Parameters

The three representative RBOs identified in Section 3.3.3, KMDL105, RD69, and Khao’ Hom Nil RBOs, were subsequently evaluated for their bone-remodeling-related activities.

#### 3.4.1. ALP Activity

**Figure 3:**
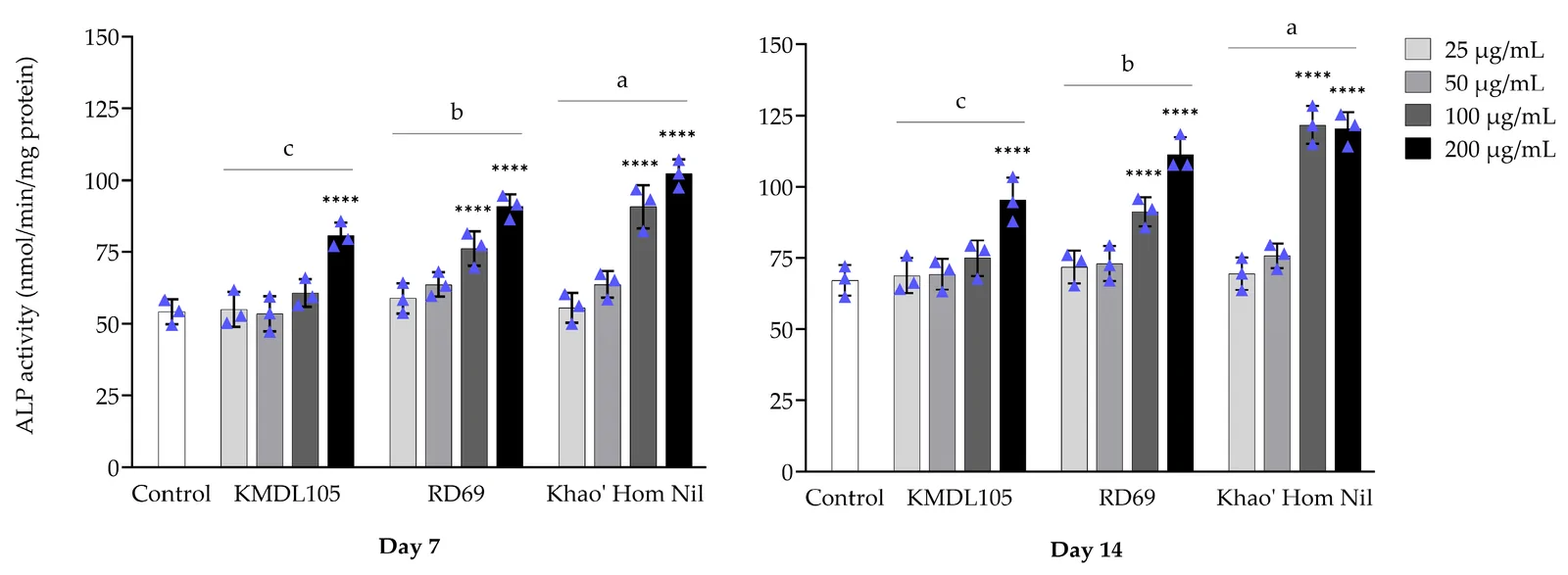
The effects of representative RBOs on ALP activity in hFOB 1.19 cells after 7 and 14 days of treatment. Cells cultured in standard culture medium without RBO treatment served as the control. Data are presented as the mean ± SD (*n* = 3). Blue triangles represent individual data points. Statistical significance was determined separately for each time point using two-way ANOVA followed by Dunnett’s multiple comparisons test for comparisons with the control group and Tukey’s multiple comparisons test for comparisons between RBO cultivars. Different lowercase letters above each cultivar indicate significant differences between RBO cultivars. **** *p* < 0.0001 compared with the control group.

The effects of representative RBOs on ALP activity in hFOB 1.19 cells after 7 and 14 days of treatment are shown in Figure 3. All representative RBOs significantly increased ALP activity compared with the control group, with the most pronounced increases generally observed at 100 and 200 µg/mL. At both time points, Khao’ Hom Nil exhibited significantly higher ALP activity than RD69 and KMDL105, whereas RD69 showed significantly higher ALP activity than KMDL105 at both time points.

#### 3.4.2. The Effects of Representative RBOs on OC, OPG, RANKL, and the RANKL/OPG Ratio

**Figure 4:**
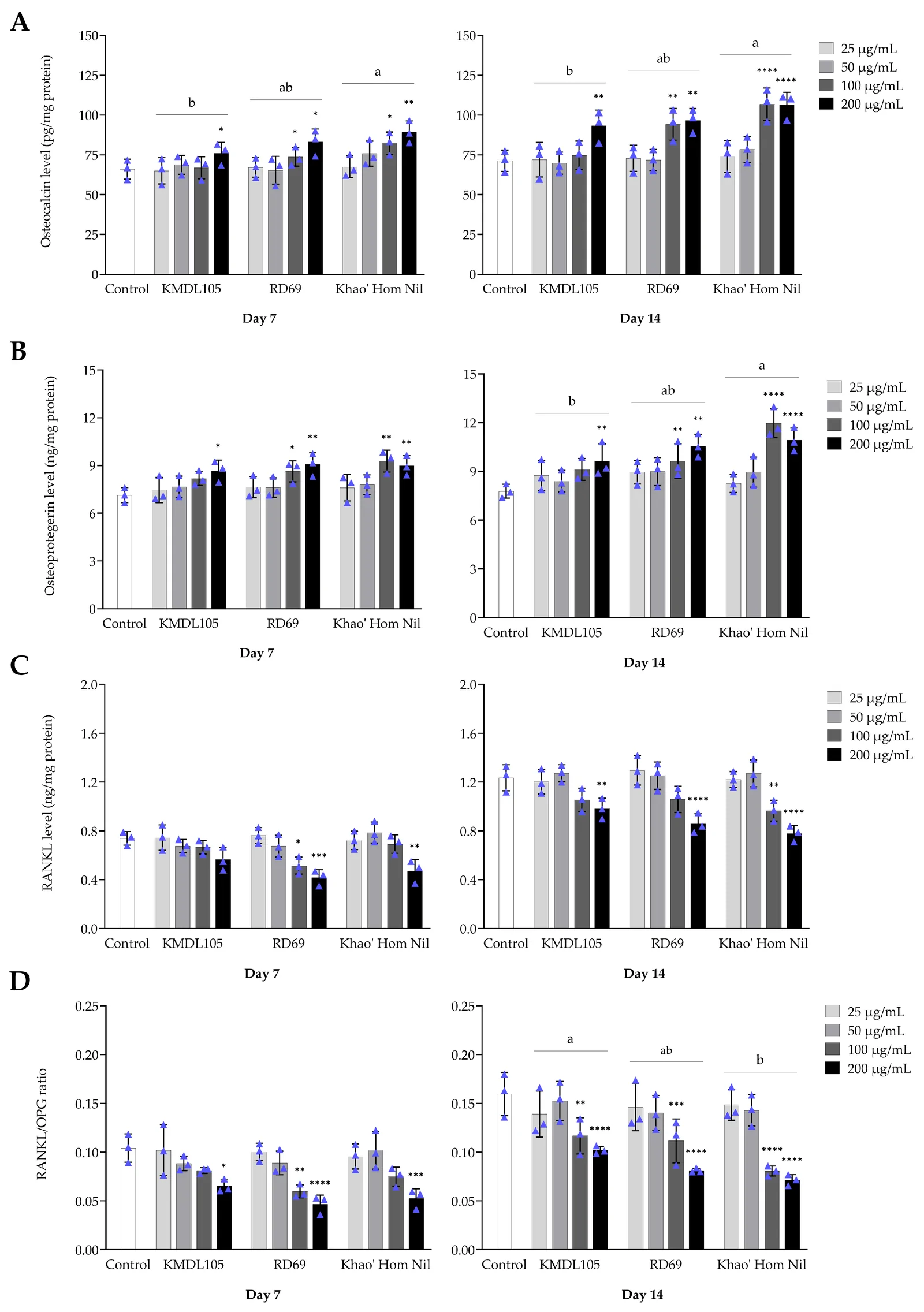
The effects of representative RBOs on (**A**) OC, (**B**) OPG, (**C**) RANKL, and (**D**) the RANKL/OPG ratio in hFOB 1.19 cells after 7 and 14 days of treatment. Cells cultured in standard culture medium without RBO treatment served as the control. Data are presented as mean ± SD (*n* = 3). Blue triangles represent individual data points. Statistical significance was determined separately for each time point using two-way ANOVA followed by Dunnett’s multiple comparisons test for comparisons with the control group and Tukey’s multiple comparisons test for comparisons between RBO cultivars. Different lowercase letters above each cultivar indicate significant differences between RBO cultivars.* *p* < 0.05, ** *p* < 0.01, *** *p* < 0.001, and **** *p* < 0.0001 compared with the control group.

The effects of representative RBOs on OC, OPG, RANKL, and the RANKL/OPG ratio in hFOB 1.19 cells after 7 and 14 days of treatment are shown in Figure 4. All representative RBOs significantly increased OC and OPG production, while significantly decreasing RANKL production and the RANKL/OPG ratio compared with the control group, with the most pronounced effects generally observed at 100 and 200 µg/mL. For OC production (Figure 4A), Khao’ Hom Nil RBO exhibited significantly higher levels than KMDL105 at both time points, whereas no significant differences were observed between Khao’ Hom Nil and RD69 or between RD69 and KMDL105. For OPG production (Figure 4B), no significant differences between cultivars were observed after 7 days of treatment. However, after 14 days, Khao’ Hom Nil exhibited significantly higher OPG production than KMDL105, while RD69 did not differ significantly from either cultivar. No significant differences in RANKL production (Figure 4C) were detected between the representative RBO cultivars at either time point. Similarly, no significant differences in the RANKL/OPG ratio were observed between cultivars after 7 days of treatment. However, after 14 days, Khao’ Hom Nil exhibited a significantly lower RANKL/OPG ratio than KMDL105, whereas RD69 did not differ significantly from either cultivar.

Among the representative RBOs, Khao’ Hom Nil and RD69 generally showed comparable, favorable trends relative to KMDL105, although only Khao’ Hom Nil reached statistical significance in most pairwise comparisons.

### 3.5. Alizarin Red S Staining

**Figure 5:**
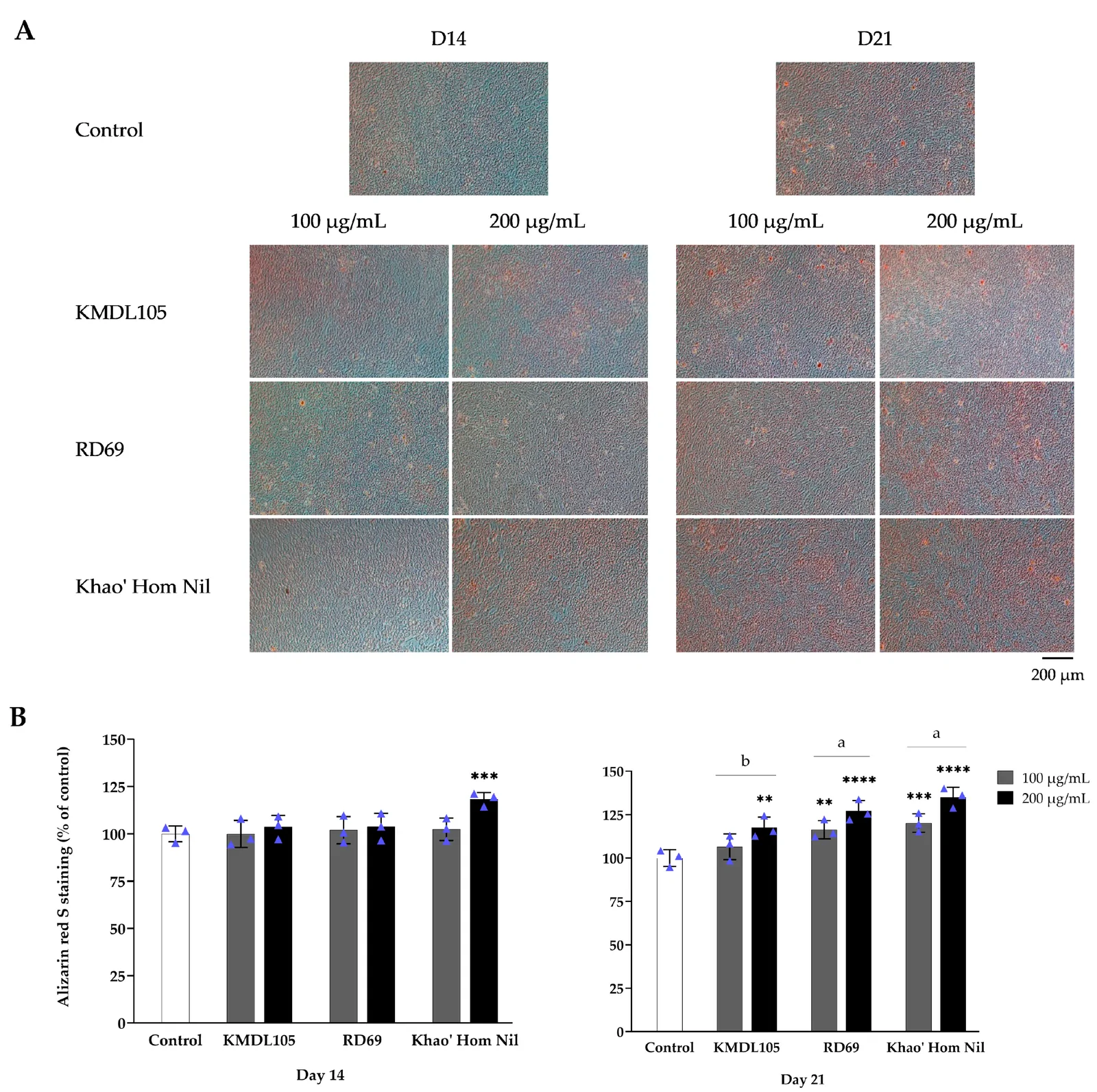
The effects of representative RBOs on matrix mineralization in hFOB 1.19 cells after 14 and 21 days of osteogenic induction. (**A**) Representative Alizarin Red S staining images of control and RBO-treated hFOB 1.19 cells at 100 and 200 µg/mL, on Day 14 and Day 21. Images were captured by light microscopy at 10× magnification; scale bar = 200 µm. (**B**) Quantitative analysis of matrix mineralization was assessed by Alizarin Red S staining, and the bound dye was quantified spectrophotometrically. Cells cultured in osteogenic differentiation medium without RBO treatment served as the control. Data are presented as the mean ± SD (*n* = 3). Blue triangles represent individual data points. Statistical significance was determined separately for each time point using two-way ANOVA followed by Dunnett’s multiple comparisons test for comparisons with the control group. Different lowercase letters above each cultivar indicate significant differences between RBO cultivars based on Tukey’s multiple comparisons test. ** *p* < 0.01, *** *p* < 0.001, and **** *p* < 0.0001 compared with the control group.

Matrix mineralization was assessed by Alizarin Red S staining (Figure 5). Representative Alizarin Red S-stained images (Figure 5A) were consistent with the quantitative analysis of matrix mineralization (Figure 5B). After 14 days of osteogenic induction, matrix mineralization was limited, with a significant increase observed only in cells treated with Khao’ Hom Nil RBO at 200 µg/mL. After 21 days, matrix mineralization increased significantly in all representative RBO-treated groups, with greater responses generally observed at 200 µg/mL. On Day 21, RD69 and Khao’ Hom Nil exhibited significantly greater matrix mineralization than KMDL105, whereas no significant difference was detected between the two pigmented RBOs.

## 4. Discussion

In the present study, pigmented RBOs generally showed slightly higher extraction yields than non-pigmented RBOs. However, extraction yield varies depending on rice cultivar and extraction conditions, and previous studies using supercritical CO_2_ extraction have reported higher oil yields in non-pigmented [38] or red rice bran oils [39], depending on cultivar. In terms of bioactive compound content, the present study demonstrated that Thai pigmented RBOs, particularly Khao’ Hom Nil and RD69, contained significantly higher levels of tocopherols, tocotrienols, and γ-oryzanol than white RBOs. Among the vitamin E isomers, γ-tocopherol and γ-tocotrienol were identified as the predominant forms in all samples. Pengkumsri et al. [40] also reported substantial levels of tocopherols and tocotrienols in pigmented RBOs, particularly black and red rice varieties. Similarly, Ng et al. [18] demonstrated that pigmented rice cultivars are generally enriched in lipophilic bioactive compounds, including tocopherols, tocotrienols, and γ-oryzanol, compared with non-pigmented cultivars, while Zhang et al. [41] reported that the lipophilic tocols fraction in black rice bran contributes more substantially to antioxidant activity than the hydrophilic anthocyanin fraction. Furthermore, Wongwaiwech et al. [38] demonstrated that oil extracted from red rice bran contains significantly higher total phytosterol content compared to that from white rice bran, supporting the concept that pigmented RBO represents a richer source of lipophilic bioactive constituents.

Oxidative stress and chronic inflammation are important contributors to bone remodeling imbalance and the progression of bone-related disorders [4,42]. Excessive production of ROS and RNS, particularly NO, has been reported to stimulate osteoclast differentiation and enhance bone resorption, thereby promoting bone loss [43,44]. In the present study, Thai pigmented RBOs exhibited greater antioxidant and anti-inflammatory activities than white RBOs by significantly suppressing intracellular ROS production in osteoblasts and reducing NO and iNOS production in activated macrophages. Among the tested samples, pigmented RBOs generally exhibited greater antioxidant and anti-inflammatory activities than the non-pigmented RBOs. These biological activities were positively correlated with the contents of tocopherols, tocotrienols, and γ-oryzanol, supporting the potential contribution of these lipophilic phytochemicals to the observed antioxidant and anti-inflammatory effects. Tocopherols and tocotrienols have been reported to scavenge free radicals, inhibit lipid peroxidation, and suppress inflammatory signaling pathways, including NF-κB activation and downstream iNOS expression [45,46]. γ-tocotrienol has been reported to protect osteoblasts against H_2_O_2_-induced oxidative stress by preventing lipid peroxidation and preserving superoxide dismutase and catalase activities [47]. The distinct phytochemical profiles together with the differential antioxidant and anti-inflammatory activities of Khao’ Hom Nil, RD69, and KMDL105 provided the rationale for selecting these representative RBOs for subsequent evaluation of bone-remodeling-related markers.

The osteogenic potential of RBOs was demonstrated by their ability to enhance ALP activity in hFOB 1.19 cells. ALP is an early marker of osteoblastic differentiation and plays an important role in bone matrix mineralization by providing inorganic phosphate required for hydroxyapatite formation [48]. In the present study, pigmented RBOs generally exhibited greater ALP-stimulating activity than non-pigmented RBOs, suggesting greater osteogenic activity during the early stage of osteoblast differentiation. Previous in vitro studies have shown that tocotrienols enhance ALP activity and promote osteogenic maturation in osteoblastic cells [33].

Osteocalcin, OPG, and RANKL are important regulators of bone remodeling and are widely used as markers of osteoblast function and osteoclastogenesis. Osteocalcin is a non-collagenous protein synthesized by mature osteoblasts and is commonly regarded as a marker of osteoblastic differentiation and bone formation, whereas OPG functions as a decoy receptor for RANKL and suppresses osteoclast differentiation by preventing RANKL–RANK interactions [49,50]. In contrast, elevated RANKL expression and an increased RANKL/OPG ratio are associated with enhanced osteoclastogenesis and bone resorption [51]. In the present study, treatment with RBOs increased osteocalcin and OPG production while reducing RANKL production and the RANKL/OPG ratio in hFOB 1.19 cells, with Khao’ Hom Nil generally exhibiting the strongest overall responses among the representative RBOs. The concurrent modulation of these markers reflects a shift toward an anti-resorptive state and a more pro-osteogenic cellular phenotype. Pigmented RBOs generally displayed more pronounced effects than the non-pigmented RBOs, a pattern that parallels their higher contents of tocopherols and tocotrienols. Whether these effects reflect the activity of specific phytochemical constituents, synergistic interactions among multiple components, or the overall phytochemical profile of the oils warrants further investigation using fractionation and mechanistic approaches. Previous preclinical evidence has highlighted tocotrienols as promising bioactive compounds for bone protection through their antioxidant and anti-inflammatory properties and their ability to modulate bone-remodeling-related processes [25]. Previous cell-based studies using hFOB 1.19 cells have demonstrated that γ- and δ-tocotrienols promote osteoblastic differentiation, as evidenced by increased alkaline phosphatase activity and osteocalcin expression [33].

The biomarker findings were further supported by the Alizarin Red S staining assay, which demonstrated increased matrix mineralization following treatment with representative RBOs. Both pigmented RBOs significantly enhanced calcium deposition and mineralized nodule formation compared with the control, with no significant difference detected between the two. The increased matrix mineralization suggests that the favorable modulation of osteogenic and bone-remodeling-related markers was accompanied by functional mineral deposition under the experimental conditions. Previous reviews have highlighted the beneficial effects of tocotrienols [25] and γ-oryzanol [29] on bone health, whereas experimental studies have demonstrated the ability of tocopherols, tocotrienols, and γ-oryzanol to promote osteoblast differentiation and bone-formation-related markers [30,33,52]. Evidence demonstrating the effects of RBO on matrix mineralization remains limited. The present findings provide additional evidence that Thai pigmented RBOs enhance matrix mineralization in an in vitro osteoblast model. Across the bone-remodeling-related markers assessed, Khao’ Hom Nil and RD69 generally showed comparable activity.

Several limitations of the present study should be acknowledged. First, only RBOs obtained by supercritical carbon dioxide extraction were investigated. Therefore, the biological activities observed in the present study may not necessarily be generalizable to RBOs prepared using other extraction methods, which can differ in extraction efficiency and phytochemical composition. Second, all biological evaluations were performed using cell-based models, which cannot fully recapitulate the complexity of bone remodeling in vivo. Third, although the present study demonstrated antioxidant, anti-inflammatory, and bone-remodeling-related activities, the underlying molecular mechanisms were not investigated, and osteoclast activity was not directly assessed. In addition, although tocopherols, tocotrienols, and γ-oryzanol are recognized as major bioactive constituents of RBO, the oil is a complex mixture containing numerous phytochemicals. Therefore, the observed biological activities may result from the combined or synergistic effects of multiple constituents, and the relative contribution of individual compounds could not be determined in the present study. Furthermore, the RBO concentrations used in the present study were selected based on preliminary cytotoxicity screening to enable comparative evaluation under controlled in vitro conditions. Therefore, these concentrations should not be directly interpreted as physiologically achievable plasma or tissue levels following dietary intake or supplementation. Future studies comparing different extraction methods, together with mechanistic investigations and in vivo models, are warranted to further clarify the biological mechanism underlying the bone-remodeling-related activities of Thai pigmented RBOs.

## 5. Conclusions

This study demonstrated that Thai pigmented RBOs, particularly Khao’ Hom Nil and RD69, contained higher levels of tocopherols and tocotrienols, and γ-oryzanol than non-pigmented RBOs. These compositional differences were associated with greater antioxidant and anti-inflammatory activities, as evidenced by reduced intracellular ROS, NO and iNOS production. In addition, representative RBOs enhanced ALP activity, increased osteocalcin and OPG production, decreased RANKL levels and the RANKL/OPG ratio, and promoted matrix mineralization in hFOB 1.19 cells. Overall, Thai pigmented RBOs represent promising natural sources of lipophilic bioactive compounds with potential antioxidant, anti-inflammatory, and bone-remodeling-related activities under in vitro conditions. Further studies comparing different extraction methods, together with mechanistic investigations and in vivo validation, are warranted to further clarify their osteoprotective potential.

## Figures and Tables

**Table 1:** Tocopherol contents in RBOs (mg/100 g oil).

Rice Bran Oil Sample	α-Tocopherol	β-Tocopherol	γ-Tocopherol	δ-Tocopherol	Total Tocopherol
KMDL105	25.93 ± 1.35 ^cd^	11.50 ± 0.60 ^cd^	64.53 ± 2.86 ^f^	7.53 ± 0.85 ^bc^	109.50 ± 2.77 ^e^
RD61	21.23 ± 1.46 ^d^	7.60 ± 1.20 ^d^	48.83 ± 1.30 ^g^	5.53 ± 1.17 ^c^	83.20 ± 1.11 ^e^
RD41	20.83 ± 1.20 ^d^	7.47 ± 0.86 ^d^	47.97 ± 2.27 ^g^	5.97 ± 0.96 ^c^	82.23 ± 2.20 ^d^
RD69	33.87 ± 1.10 ^a^	19.23 ± 1.03 ^a^	142.73 ± 3.71 ^c^	14.70 ± 1.11 ^a^	210.53 ± 4.13 ^a^
Red Jasmin Rice	27.57 ± 0.67 ^bc^	13.23 ± 0.86 ^bc^	113.70 ± 5.74 ^e^	11.33 ± 1.20 ^ab^	165.83 ± 3.95 ^c^
Sang Yod	31.90 ± 0.95 ^a^	17.47 ± 0.74 ^a^	139.00 ± 2.36 ^c^	14.33 ± 1.39 ^a^	202.70 ± 3.93 ^ab^
Khao’ Hom Nil	35.83 ± 1.27 ^a^	22.33 ± 0.97 ^a^	161.10 ± 3.24 ^a^	15.50 ± 1.28 ^a^	234.77 ± 3.07 ^a^
Riceberry	33.27 ± 0.85 ^a^	18.50 ± 1.20 ^a^	138.77 ± 2.73 ^c^	14.00 ± 1.05 ^a^	204.53 ± 1.22 ^ab^
CMU107	30.67 ± 1.11 ^ab^	17.23 ± 0.71 ^ab^	129.13 ± 3.31 ^d^	13.60 ± 0.90 ^a^	190.63 ± 4.54 ^b^

Values are presented as mean ± SD (*n* = 3). Different superscript letters within the same column indicate significant differences between samples at *p* < 0.05.

**Table 2:** Tocotrienol contents in RBOs (mg/100 g oil).

Rice Bran Oil Sample	α-Tocotrienol	β-Tocotrienol	γ-Tocotrienol	δ-Tocotrienol	Total Tocotrienols
KMDL105	13.03 ± 0.70 ^bc^	ND	88.17 ± 5.34 ^e^	8.47 ± 1.19 ^b^	109.67 ± 4.86 ^e^
RD61	8.60 ± 0.82 ^c^	ND	58.23 ± 3.69 ^f^	5.80 ± 0.90 ^b^	72.63 ± 4.27 ^f^
RD41	8.67 ± 0.81 ^c^	ND	48.40 ± 2.26 ^g^	6.20 ± 0.79 ^b^	63.27 ± 2.93 ^f^
RD69	24.40 ± 1.11 ^a^	ND	175.40 ± 3.63 ^a^	15.60 ± 1.30 ^a^	215.40 ± 5.05 ^a^
Red Jasmin Rice	14.33 ± 1.34 ^b^	ND	113.70 ± 5.74 ^d^	11.43 ± 0.65 ^ab^	139.47 ± 5.10 ^d^
Sang Yod	26.03 ± 1.60 ^a^	ND	158.17 ± 5.25 ^b^	13.63 ± 0.83 ^ab^	197.83 ± 5.58 ^b^
Khao’ Hom Nil	24.87 ± 1.46 ^a^	ND	199.43 ± 6.73 ^a^	17.37 ± 1.06 ^a^	241.67 ± 9.00 ^a^
Riceberry	22.70 ± 1.01 ^a^	ND	163.57 ± 5.34 ^b^	13.70 ± 0.92 ^ab^	199.97 ± 5.06 ^b^
CMU107	18.53 ± 1.25 ^ab^	ND	143.63 ± 6.37 ^c^	13.80 ± 1.35 ^ab^	175.97 ± 6.67 ^c^

Values are presented as mean ± SD (*n* = 3). Different superscript letters within the same column indicate significant differences between samples at *p* < 0.05. ND, not detected.

**Table 3:** γ-Oryzanol contents in RBOs (mg/g oil).

Rice Bran Oil Sample	γ-Oryzanol
KMDL105	6.17 ± 0.19 ^g^
RD61	6.15 ± 0.12 ^g^
RD41	5.83 ± 0.07 ^h^
RD69	9.76 ± 0.10 ^a^
Red Jasmin Rice	7.48 ± 0.15 ^f^
Sang Yod	8.70 ± 0.14 ^d^
Khao’ Hom Nil	9.11 ± 0.12 ^b^
Riceberry	8.90 ± 0.15 ^c^
CMU107	7.80 ± 0.12 ^e^

Values are presented as mean ± SD (*n* = 3). Different superscript letters within the same column indicate significant differences between samples at *p* < 0.05.

## Data Availability

The data supporting the findings of this study are available from the corresponding author upon reasonable request.

## Supplementary Materials

The following supporting information can be downloaded from: https://www.mdpi.com/article/10.3390/antiox15080981/s1. Table S1. Extraction yields of rice bran oils (RBOs) obtained by supercritical CO_2_ extraction from nine Thai rice cultivars; Table S2. Analytical performance of the HPLC methods for the determination of representative tocopherols, tocotrienols, and γ-oryzanol, calculated according to the ICH Q2(R1) guideline [53]; Figure S1. Representative RP-HPLC chromatograms of tocopherol and tocotrienol standards and a rice bran oil (RBO) sample; Figure S2. Representative RP-HPLC chromatograms of the γ-oryzanol standard and a representative RBO sample; Figure S3. The effects of RBOs on cell viability in RAW264.7 and hFOB 1.19 cells after 72 h of treatment; Table S3. Pearson correlation analysis between major bioactive compounds (tocopherols, tocotrienols, and γ-oryzanol) and intracellular ROS, NO, and iNOS production.

## Abbreviations

The following abbreviations are used in this manuscript:

ALP	alkaline phosphatase
DAF-FM DA	4-amino-5-methylamino-2′,7′-difluorofluorescein diacetate
DCF	2′,7′-dichlorofluorescein
DMEM	Dulbecco’s modified Eagle medium
FBS	fetal bovine serum
H_2_DCF-DA	2′,7′-dichlorodihydrofluorescein diacetate
HPLC	high-performance liquid chromatography
IFN-γ	interferon-gamma
iNOS	inducible nitric oxide synthase
LPS	lipopolysaccharide
MAPK	mitogen-activated protein kinase
NAC	n-acetylcysteine
NF-κB	nuclear factor kappa-b
NO	nitric oxide
OC	osteocalcin
OPG	osteoprotegerin
PBS	phosphate-buffered saline
*p*NP	*p*-nitrophenol
*p*NPP	*p*-nitrophenyl phosphate
RANKL	receptor activator of nuclear factor kappa-b ligand
RBO	rice bran oil
RNS	reactive nitrogen species
ROS	reactive oxygen species
SFE	supercritical fluid extraction
TNF-α	tumor necrosis factor-alpha
