# Supplementary material for: Antioxidant, Anti-Inflammatory, and Bone-Remodeling Modulatory Activities of Thai Pigmented and Non-Pigmented Rice Bran Oils Containing Tocols and γ-Oryzanol
Source: Antioxidants (Basel). 2026 Aug 7;15(8):981. doi: 10.3390/antiox15080981 (PMC13509597; doi:10.3390/antiox15080981)
Supplement: Supplementary file 1 [file antioxidants-15-00981-s001.zip › antioxidants-4392313-supplementary.pdf]

antioxidants

MDPI

Article

Antioxidant, Anti-Inflammatory, and Bone-Remodeling Modulatory Activities of Thai Pigmented and Non-Pigmented Rice Bran Oils Containing Tocols and γ-Oryzanol

Thanawat Pattananandecha [1,2]*, Sutasinee Apichai [1,2], Jakaphun Julsrigival [2,3], Kannika Thiankhanithikun [2,4], Fumihiko Ogata [5], Naohito Kawasaki [5,6], Jetsada Ruangsuriya [2,7], and Chalermpong Saenjum [2,3]*

[1] Office of Research Administration, Chiang Mai University, Chiang Mai 50200, Thailand; thanawat.pdecha@gmail.com (T.P.); sutasinee.apichai@gmail.com (S.A.)
[2] Research Center for Innovation in Analytical Science and Technology for Biodiversity-Based Economic and Society (I-ANALY-S-T_B.BES-CMU), Chiang Mai University, Chiang Mai 50200, Thailand; jakaphun.jul@cmu.ac.th (J.J.); kannika.th@cmu.ac.th (K.T.); jetsada.ruang@cmu.ac.th (J.R.); chalermpong.s@cmu.ac.th (C.S.)
[3] Department of Pharmaceutical Sciences, Faculty of Pharmacy, Chiang Mai University, Chiang Mai 50200, Thailand
[4] Department of Pharmaceutical Care, Faculty of Pharmacy, Chiang Mai University, Chiang Mai 50200, Thailand
[5] Faculty of Pharmacy, Kindai University, 3-4-1 Kowakae, Higashi-Osaka, Osaka 577-8502, Japan; ogata@phar.kindai.ac.jp (F.O.); kawasaki@phar.kindai.ac.jp (N.K.)
[6] Antiaging Center, Kindai University, 3-4-1 Kowakae, Higashi-Osaka, Osaka 577-8502, Japan
[7] Department of Biochemistry, Faculty of Medicine, Chiang Mai University, Chiang Mai 50200, Thailand
* Correspondence: chalermpong.s@cmu.ac.th; Tel +66 89 950 4227, thanawat.pdecha@gmail.com; Tel +66 93 146 3249

Table S1. Extraction yields of rice bran oils (RBOs).

Rice bran oils	Extraction yield (%, w/w)
KMDL105	12.86 ± 0.90[bc]
RD61	11.75 ± 1.27[c]
RD41	12.89 ± 1.19[bc]
RD69	14.30 ± 0.47[a]
Red Jasmin Rice	13.25 ± 0.58[b]
Sang Yod	13.40 ± 0.59[b]
Khao' Hom Nil	14.31 ± 0.53[a]
Riceberry	14.32 ± 0.39[a]
CMU107	13.03 ± 0.96[bc]

Values are presented as mean ± SD (n = 3). Different superscript letters within the same column indicate significant differences between samples at $p < 0.05$.

Table S2. Analytical performance of the HPLC methods for the determination of representative tocopherols, tocotrienols, and γ-oryzanol.

Analyte	Linearity range (µg/mL)	R^2	LOD (µg/mL)	LOQ (µg/mL)	Mean %RSD
**Tocols**					
α-tocopherol	5–50	0.9983	1.72	5.22	3.98
β-tocopherol	5–50	0.9987	1.45	4.40	4.39
γ-tocopherol	5–50	0.9994	0.97	2.94	3.84
δ-tocopherol	5–50	0.9988	1.34	4.07	2.90
α-tocotrienol	5–50	0.9987	1.53	4.64	4.50
β-tocotrienol	5–50	0.9991	1.22	3.69	2.23
γ-tocotrienol	5–50	0.9987	1.46	4.43	3.77
δ-tocotrienol	5–50	0.9992	1.09	3.31	2.01
**γ-oryzanol**					
Cycloartenyl ferulate	20–200	0.9984	6.24	18.92	1.09
24-Methylenecycloartanyl ferulate	15–200	0.9989	5.22	15.81	1.55
Campesteryl ferulate	15–200	0.9989	5.13	15.54	1.66
β-Sitosteryl ferulate	15–200	0.9989	5.16	15.64	1.48

LOD, limit of detection; LOQ, limit of quantification; R^2, coefficient of determination; %RSD, percentage relative standard deviation. LOD and LOQ were calculated according to the ICH Q2(R1) guideline [1].

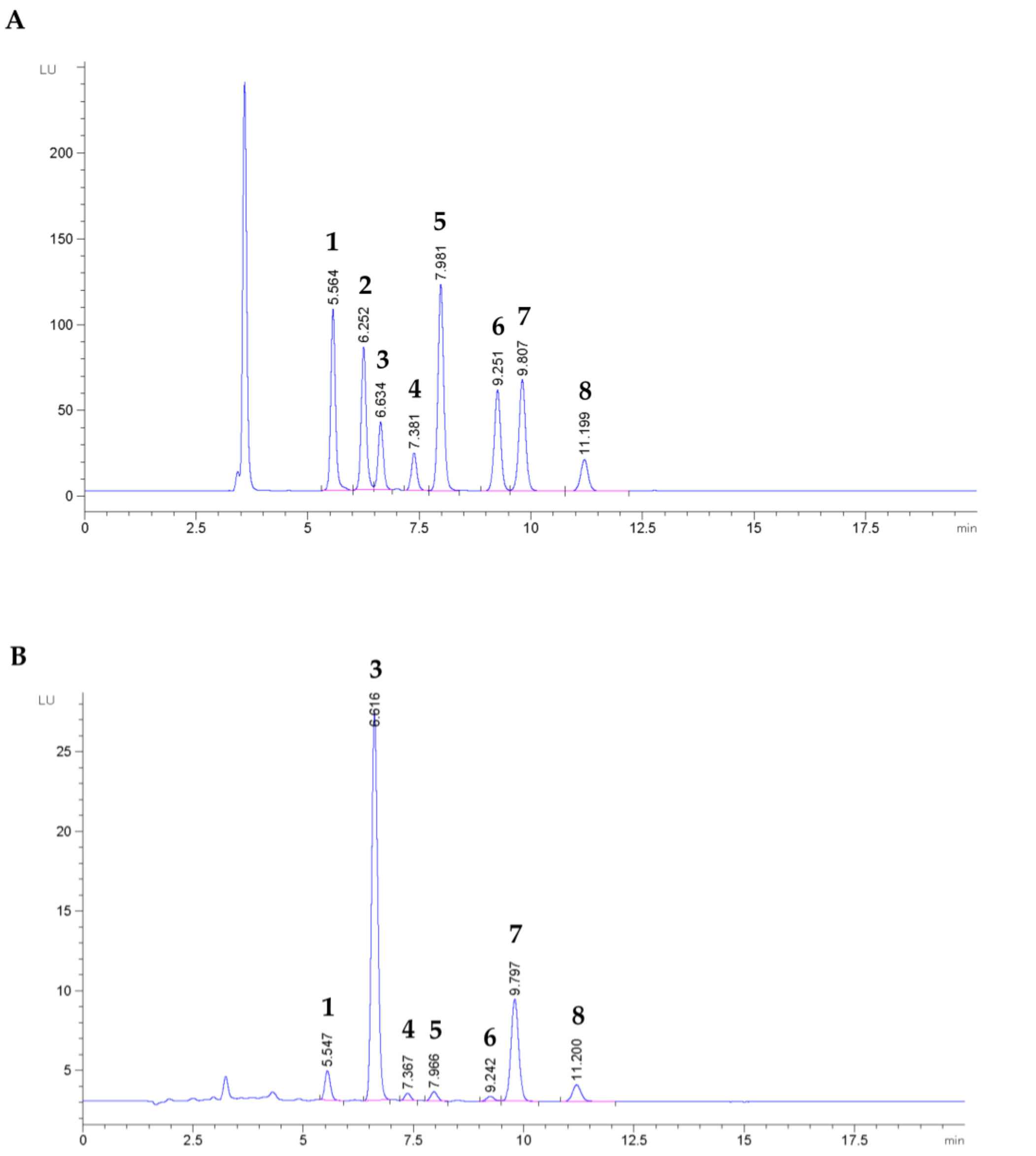

Figure S1. Representative RP-HPLC chromatograms of tocopherols and tocotrienols: (A) mixed tocopherol and tocotrienol standards and (B) representative RBO sample. Peak assignments: (1) δ-tocotrienol, (2) β-tocotrienol, (3) γ-tocotrienol, (4) α-tocotrienol, (5) α-tocopherol, (6) β-tocopherol, (7) γ-tocopherol, and (8) δ-tocopherol.

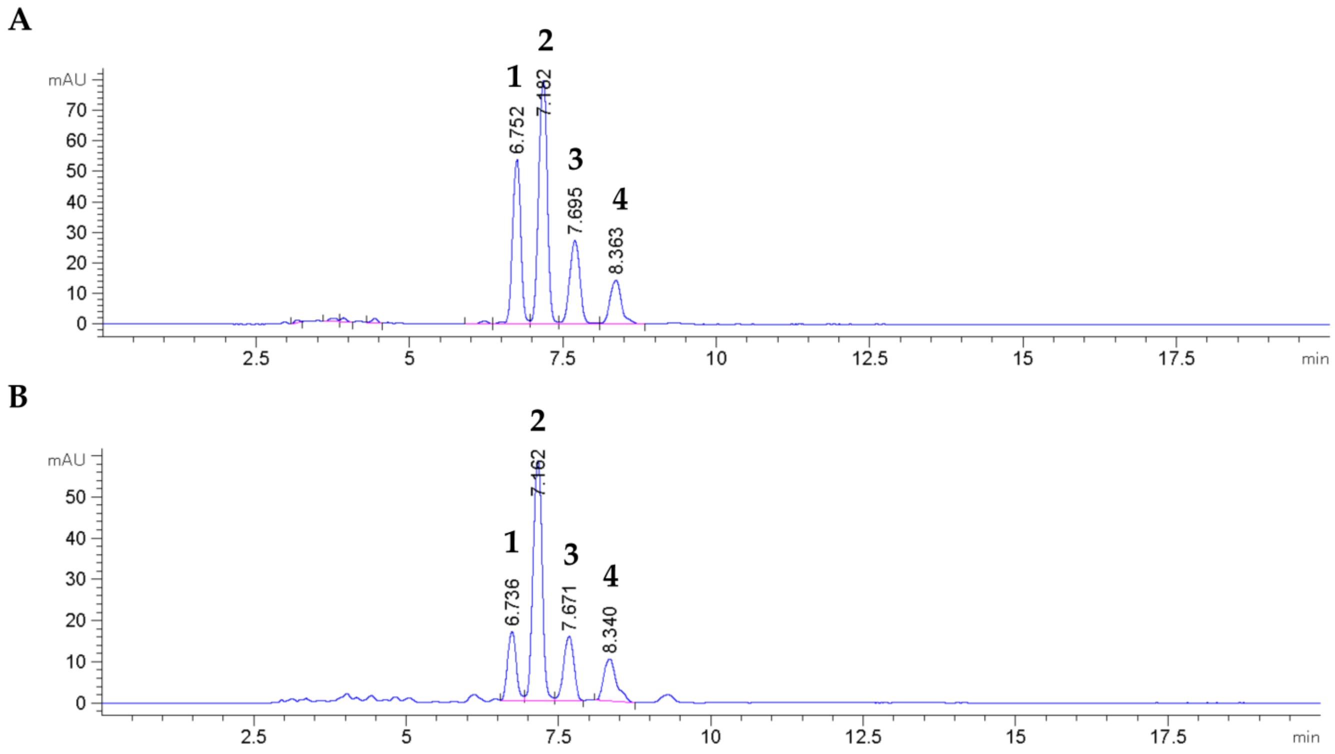

Figure S2. Representative RP-HPLC chromatograms of the γ-oryzanol: (A) γ-oryzanol standard and (B) representative RBO sample. Peak assignments: (1) cycloartenyl ferulate, (2) 24-methylenecycloartanyl ferulate, (3) campesteryl ferulate, and (4) β-sitosteryl ferulate.

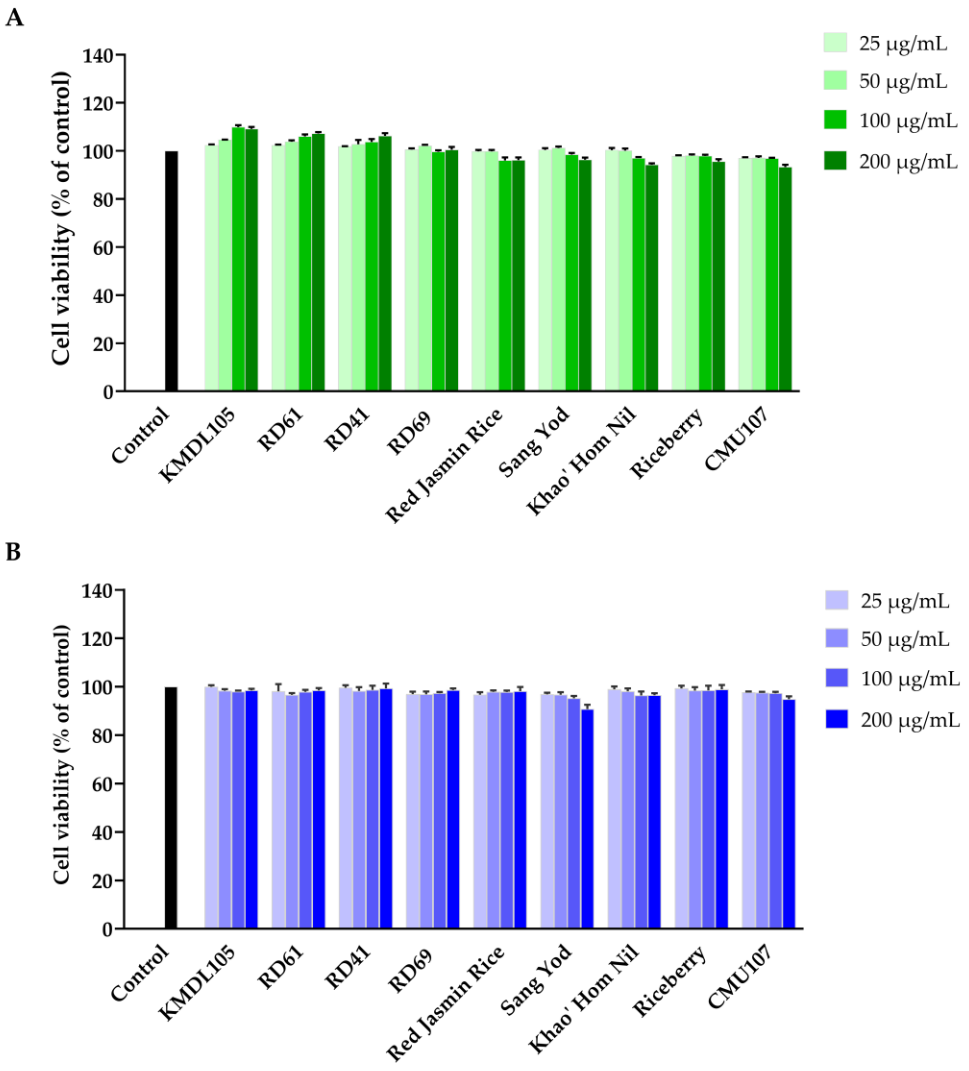

Figure S3. The effects of RBOs on cell viability in (A) RAW264.7 cells and (B) hFOB 1.19 cells following 72 h of treatment. Data are presented as mean ± SD (n = 3).

Table S3. Pearson correlation coefficients between total tocopherol, total tocotrienol and γ-oryzanol contents and intracellular ROS, NO and iNOS production in RBOs.

Variable	ROS			NO			iNOS		
	r	95% CI	p	r	95% CI	p	r	95% CI	p
Total tocopherol	-0.983	-0.9966 to -0.9188	0.0001	-0.931	-0.9857 to -0.6988	0.0001	-0.916	-0.9825 to -0.6442	0.0001
Total tocotrienol	-0.987	-0.9973 to -0.9360	0.0001	-0.958	-0.9914 to -0.8076	0.0003	-0.958	-0.9913 to -0.8056	0.0005
γ-oryzanol	−0.946	−0.9890 to −0.7604	0.0001	−0.886	−0.9759 to −0.5385	0.0015	−0.896	−0.9781 to −0.5725	0.0011

Data were analyzed using Pearson's correlation analysis (n = 9). Correlation coefficients (r), 95% confidence intervals (CI), and two-tailed p values are presented. Correlation analysis was performed using data obtained at 200 μg/mL of RBOs.

References

1. International Council for Harmonisation (ICH) *Validation of Analytical Procedures: Text and Methodology Q2(R1)*; ICH Secretariat: Geneva, Switzerland, 2005.
